# Correspondence

**Published:** 1856-01

**Authors:** N. B. Slayton

**Affiliations:** Madison, Indiana


Madison, Indiana, Dec. 10, 1855.

Mr. Editor—Sir, You will do me a favor, and I doubt not confer the same on many of the profession, if you can spare room in your Journal to insert a few lines on the proper use of gutta percha as a base for artificial teeth.

Some few weeks since I sent a small circular to as many of the profession as I could learn of their whereabouts, giving the result of a course of experiments that I had made with gutta percha. I had no idea at that time that it would attract so much attention, or be received so favorably by the profession. I then offered it to the profession for temporary work, and for nothing more, and that is all I now claim for it. I stated at the same time that I was satisfied that in many cases it might be used to advantage for permanent work, and from my experience since that time, as I became better acquainted with the properties of gutta percha, and the manner of working it, my impression is, that in nearly every case where the teeth are long, and gums soft and tender, it will be preferred to any other material for permanent work, both by the patient and dentist.

Gutta percha cannot be used for *every* thing, neither can it be used to advantage in every case, *even for temporary work.* I wish here to state where gutta percha *can*, and can not be used to advantage, so far as my own experience goes. *Others* may claim what they choose.

Where teeth are extremely short and set close together, you have not the room to unite the gutta percha through and around them, so as to make it firm and durable. If short teeth were made with narrow necks, so as to let the gutta percha pass freely through and unite firmly on the other side, it might do well. The teeth that are best adapted to this kind of work, are those made for Allen's continuous gum work. They have narrow necks, and will permit the gutta percha to pass freely between them, and unite firmly around the teeth, and at the same time adhere firmly to the gutta percha on the opposite side. This is necessary both for strength and neatness, and if properly done, will prevent any food from lodging around the teeth, or even moisture getting between them and the gutta percha, thereby preventing any unpleasant smell that would naturally arise from it. Common plate teeth will do well, if ground narrow at the base. It is as necessary for a dentist to exercise his judgment in the working of gutta percha, as in any other part of his duties. It is impossible to stick gutta percha on plate, or on the teeth, and have it remain long, unless a man understands well the manner of working it, and then it requires judgment to know when and where it should be used.

I have spent a good deal of time and money in learning the best way of working gutta percha, and to make it as simple as possible. I don't pretend to have perfected the working of this material, but one thing I do say, that unless a dentist is well posted up in the manner of working gutta percha, he will find it cheaper for him to pay any reasonable sum for that instruction, than to learn it from his own experience; that is, if he values his time worth anything, throwing aside the expense and trouble.

I have within the last six weeks instructed a large number of dentists, some work it beautifully, while others make a perfect botch of it. It is not only necessary for a man to see it done, but he must take hold and do it himself, and if he should fail the first time *no matter*, *try again*. The only way this gutta percha can be brought to that perfection, that our profession needs, is for every one to try and make some little improvements, and when these are all combined, I doubt not we shall have just the thing we want. My greatest fears are, that the profession as a *mass* are expecting too much of this new material, and will use and recommend it when they ought not to, and thereby kill it in the bud. I find dentists are like every body else, too much inclined to make a hobby of a new thing, and use it in every case, and recommend it to *all* their patients as being superior to every thing else; and then if it should not prove what *they themselves* claim for it, will condemn it as being a *great humbug*. Such has been the fate of some of our most valuable improvements, and such *may* be the fate of gutta percha, if not used with more judgment than some are now doing. My intention is to try and bring this improvement within the reach of every dentist, but not to force it upon any one. I don't think it possible for me to explain the manner of working gutta percha, on paper, so that a dentist can work it successfully without further instruction, unless he is well posted up on its peculiar properties, and has experimented with it before; such is the opinion of nearly all when they have once seen it worked.

I am not selling a patent right. If a dentist wishes to learn my manner of putting up this style of work, I instruct him, or cause my agent to do so, and charge him a reasonable price for my services.

On my first bringing this before the profession, I made application for a patent, for my mode of working gutta percha, but by the advice of a number of dentists, who thought it might prejudice many against it, I withdrew that application, and secured myself on the gutta percha; that is, my manner of *refining* and *coloring* it, and then sell no man the material, unless he has first been instructed how to use it. In this way I secure myself, and give them that have received instructions better security than I could by a patent. I have several agents in the United States who are authorized to instruct dentists in the manner of doing this work, and they will try and bring it within the reach of all. I have made Messrs. Jones, White and McCurdy of New York and Philadelphia, my only agents for the manufacture and sale of the colored gutta percha, and from the well known reputation of this house, I think the profession may rest assured that they will receive nothing from their hands but what is pure, and that they will do the best to make every improvement that is possible to make with gutta percha for dental purposes.

The cost of the gutta percha for a full upper set of teeth, is from seventy-five cents to one dollar and fifty cents, with no waste, and the time usually spent in making them is about four hours, and when properly done will wear smooth and hard. It can be made as firm as any plate work, and look as well as the best gum teeth. To keep this work clean and sweet all that is necessary is to give your patient a suitable brush, and have him use soap and water freely.

I have deemed it necessary to make these explanations, that any one may see what I *do claim*, and what I *do not*, and how the gutta percha is disposed of, the cost of material, and who it can be obtained from.

Any one wishing further information on this subject, I will give it cheerfully.

Your's respectfully,

N. B. SLAYTON.

*Madison, Indiana.*

